# Retention order prediction of peptides containing non-proteinogenic amino acids

**DOI:** 10.1093/bioadv/vbaf246

**Published:** 2025-10-08

**Authors:** Shohei Nakamukai, Eisuke Hayakawa, Tetsuya Mori, Yuji Ise, Masami Yokota Hirai, Masanori Arita

**Affiliations:** RIKEN Center for Sustainable Resource Science, Yokohama, Kanagawa 230-0045, Japan; RIKEN Center for Sustainable Resource Science, Yokohama, Kanagawa 230-0045, Japan; Department of Bioscience and Bioinformatics, School of Computer Science and Systems Engineering, Kyushu Institute of Technology, Iizuka, Fukuoka 820-8502, Japan; RIKEN Center for Sustainable Resource Science, Yokohama, Kanagawa 230-0045, Japan; Fujitsu Research, Fujitsu Limited, Kanagawa 211-8588, Japan; Department of Human Environmental Studies, Hiroshima Shudo University, Hiroshima 731-3195, Japan; RIKEN Center for Sustainable Resource Science, Yokohama, Kanagawa 230-0045, Japan; RIKEN Center for Sustainable Resource Science, Yokohama, Kanagawa 230-0045, Japan; National Institute of Genetics, Mishima, Shizuoka 411-8540, Japan

## Abstract

**Motivation:**

Peptides containing non-proteinogenic amino acids (PNPAs) are promising targets in drug development for their unique pharmacological properties. The lack of their mass spectra or retention data has been hindering PNPA research, where accurate assessment of their retention time in chromatography is crucial for identifying structures and characterizing functions. Conventional methods are often ineffective due to limited amount of data. This study aims to predict their retention order, not absolute time, from structures by using data from peptides and small molecules. This approach can advance natural product identification and drug research.

**Results:**

Our model uses the Ranking Support Vector Machine, and successfully predicted the retention order of PNPA with an accuracy of over 0.9. Counting fingerprints and MIX fingerprint, which combines four types of fingerprints, were used as explanatory variables. To suppress the multi-collinearity, principal component analysis was applied to reduce spurious fingerprints. SHAP value analysis revealed that one component, derived from methyl groups, contributed most for the prediction. Overall, order prediction can effectively find candidate compounds from LC/MS data from non-conventional biological extracts.

**Availability and implementation:**

https://github.com/ShoheiNakamukai/RO_prediction_of_PNPA/tree/main.

## 1 Introduction

Metabolome analysis aims at comprehensive identification of compounds in biological samples through measurements with liquid chromatography- or gas chromatography mass spectrometry (LC/MS or GC/MS) (Stein 1999, van der Hooft *et al.* 2013, Tsugawa *et al.* 2015). Among various metabolome, identification of marine natural products is challenging, due to their limited amount of information in major spectral databases. For instance, Zhang *et al.* could identify only 6.7% of compounds in marine sponges (Zhang *et al.* 2022), compared to 18.5% in plant extracts (Tsugawa *et al.* 2019) via the tandem MS. The lack of information is largely due to the difficulty in collecting natural products; many marine organisms are unculturable and their biosynthetic products often exist in trace amounts (Molinski *et al.* 2009). Among such unique structures with pharmacological activities, our focus is marine peptides that contain non-proteinogenic amino acids, called here as PNPAs (Desriac *et al.* 2013). PNPAs share synthetic methodologies and MS/MS analyses with other standard peptides (Shinbara *et al.* 2020, Zervou *et al.* 2020), but non-proteinogenic amino acids may contain numerous modifications and circular structures with different stereochemistry. For this reason, prediction of their retention time remains challenging for existing tools such as Prosit or DeepLC (Gessulat *et al.* 2019, Bouwmeester *et al.* 2021), whose background models are trained on canonical proteinogenic amino acids without consideration of isobaric structure or complex modifications beyond common methylation or oxidation.

Prediction of retention time is platform-dependent; different chromatographic conditions result in different retention times. On the other hand, Stanstrup *et al.* have shown that retention order is largely conserved across systems with similar C18 stationary phases, even when different organic eluents or gradients are used (substantial difference, such as the use of acidic *versus* alkaline eluents or C8-based columns, affects retention order) (Stanstrup *et al.* 2015). Prediction of the retention order is more practical than the prediction of absolute time, and the Ranking Support Vector Machine (RankSVM) was proposed for predicting the ordering of small molecules with high accuracy (Bach *et al.* 2018).

**Figure 1.**
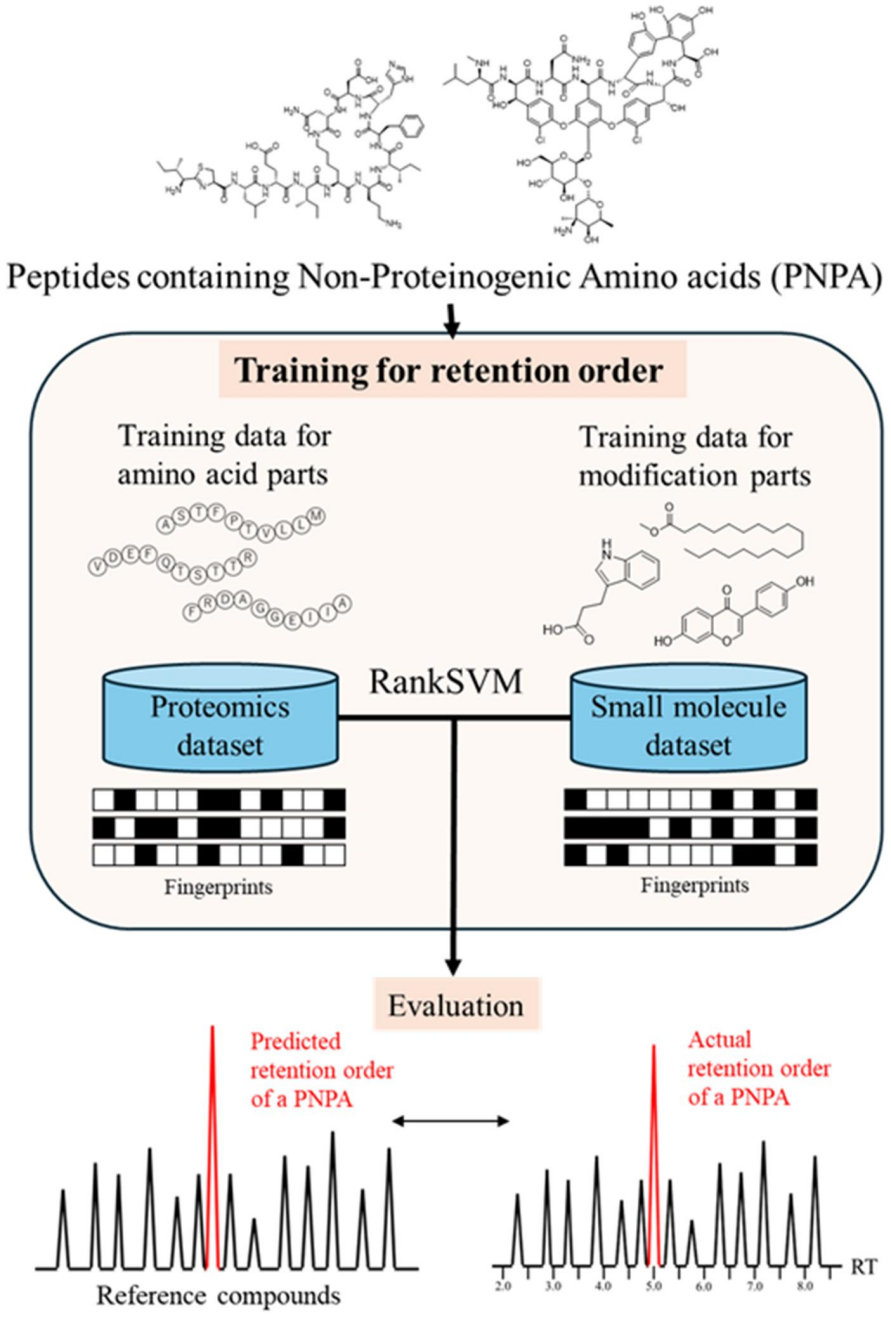
Overview of the evaluation of retention order prediction of PNPA.

In this study, we applied this RankSVM prediction to the LC/MS data of PNPAs. Major difficulty is the scarcity of MS and retention time information on PNPAs; large datasets are not available in contrast to small molecules. We therefore utilized peptide data for training the amino acid components together with small-molecule data for training the molecular modifications. The prediction model was evaluated by comparing the predicted retention order of PNPAs to reference compounds with known retention time (Fig. 1). As a proof of concept, we applied this model for the analysis of marine sponge extracts. Using precursor *m*/*z* values obtained from LC/MS measurements and retention order prediction, we were able to effectively narrow down candidate structures from structure databases for a compound in the sponge samples. This demonstrates that the integration of both *m*/*z* and retention order information enables efficient structure annotation. This approach exemplifies a new method to identify underrepresented molecular types such as PNPAs by circumventing the limitations of current MS/MS databases in metabolomics.

## 2 Methods

### 2.1 Data acquisition of LC-MS retention time

We selected four datasets from the PredRet database (http://predret.org/): Eawag_XBridgeC18 (317 compounds), FEM_long (281 compounds), RIKEN (181 compounds), and UFZ_Phenomenex (192 compounds).

These datasets contain flavonoids, alkaloids, terpenoids, amino acids, glucosinolates, and others. The four sets were combined and named “PredRet.”

The mass RAW data of peptides and single-cell RNA sequencing data were downloaded from the ProteomeXchange Consortium with the dataset identifier PXD030145 (Hayakawa *et al.* 2022). The sequence and retention time of 787 peptides were extracted. The peptides were sorted in order of retention time, and they were sequentially assigned into five subsets (Nematostella_1, Nematostella_2, Nematostella_3, Nematostella_4, and Nematostella_5) in such a way that the retention time distribution became uniform among each group. The dataset containing these five subsets was named “Nematostella”. The description for these two datasets is detailed in Supporting Information, available as supplementary data at *Bioinformatics Advances* online.

### 2.2 Measurement of standard compounds

Total 22 compounds and 9 PNPA standards covering a long time-range were purchased as reference standards (Table 1). Each standard compound was experimentally measured with our in-house LC/MS platform with conditions in Supporting Table 1, available as supplementary data at *Bioinformatics Advances* online.

**Table 1.** List of reference compounds and peptides as the test dataset and their retention times (RT).

Reference compounds	CAS number	RT
2-Propenyl glucosinolate	534-69-0	1.74
p-Hydroxy benzyl glucosinolate	20196-67-2	2.02
Cyanidin 3-O-sambubioside 5-O-glucoside	53925-33-0	2.52
Carbazochrome sulfonate	70063-04-6	2.70
Sinomenine	115-53-7	3.04
(-)-Epicatechin	490-46-0	3.49
7,8-Dihydroxy coumarin	486-35-1	3.69
Isovitexin	29702-25-8	4.03
3-Hydroxy cinnamic acid	588-30-7	4.47
Coniferyl aldehyde	458-36-6	4.81
3,4-Dimethoxy cinnamic acid	2316-26-9	5.23
Quercetin	117-39-5	5.48
4-Methoxy cinnamic acid	943-89-5	5.90
2-Methoxy cinnamic acid	1011-54-7	6.16
Isoliquiritigenin	961-29-5	6.51
6-Hydroxy flavanone	4250-77-5	7.19
2'-Hydroxy flavanone	17348-76-4	7.48
Atractylenolide III	73030-71-4	7.98
trans-Pterostilbene	537-42-8	8.07
Triacetyl resveratrol	42206-94-0	8.51
Magnolol	528-43-8	9.22
Corosolic acid	4547-24-4	9.95

PNPA	CAS number	RT
Vancomycin	1404-93-9	2.68
[Arg8]-Vasopressin	113-79-1	3.11
α-Amanitin	23109-05-9	3.41
Bacitracin	1405-87-4	4.67
Daptomycin	103060-53-3	6.56
Epothilone B	152044-54-7	7.67
Gramicidin A	11029-61-1	9.19
Actinomycin D	50-76-0	9.54
Cyclosporine A	59865-13-3	10.93

### 2.3 Molecular fingerprinting and principal component analysis

A binary fingerprint represents an array of existence/absence of specified molecular substructures, and a counting fingerprint represents an array of occurrence of molecular substructures. In this study, MACCS, extended standards, graph, and circular fingerprints were calculated using the rcdk package (Guha 2007) from molecular structures without stereo specificity. The SMARTS (SMILES Arbitrary Target Specification) patterns for MACCS fingerprints, which are 166 predefined substructure keys, were the same as the previous study (Bach *et al.* 2018). These four types of fingerprints were combined and referred to as the MIX fingerprint. Then, Principal Component Analysis (PCA) was applied to the MIX fingerprint of the training datasets, which included peptide data, small molecules, and reference compounds. The purpose of this PCA was to identify correlated bit strings, reduce multicollinearity, simplify the model, and improve its performance (Ji *et al.* 2020). The resulting processed fingerprint dataset was called principal component fingerprint (PCF). The transformation matrices obtained from the PCA applied to the training datasets were reused to transform the test datasets, to ensure consistency between the datasets. Details of these calculations are given in Supporting Information, available as supplementary data at *Bioinformatics Advances* online.

### 2.4 Prediction of retention order with ranking support vector machine (RankSVM)

The Ranking Support Vector Machine (RankSVM) was employed to predict the retention order of molecular pairs (Bach *et al.* 2018). In this method, all pairs of compounds within the same dataset were first generated, and their RT values were compared. Each pair was labeled as either “correct” or “reverse” based on the difference in their RT values. In RankSVM, cross-validation was employed to optimize the model’s performance and to ensure its prediction ability. The training was conducted in the following three patterns: (i) PredRet using only small molecules as training data (4 subsets from the PredRet database); (ii) Nematostella using only peptide data as training data (5 subsets from the Nematostella); and (iii) PredRet + Nematostella using both small molecules and peptide data (4 + 5 subsets). For all three patterns, a subset of reference compounds was added. Afterward, training was conducted for each subset, followed by cross-validation using all subsets.

### 2.5 Evaluation of retention order prediction model

For each PNPA compound, its retention order relative to the *i*th reference compound was described as *m*_*i*_ and *p*_*i*_ for measured and predicted values, respectively. The prediction accuracy *S* for each PNPA was calculated to assess the degree of agreement between the measured retention order *m*_*i*_ and the predicted retention order *p*_*i*_ as the following.


(mi, pi) = { 1 (mi=pi) 0 (mi≠ pi)

$$S=\;\frac{1}{n}\sum_{i\;=\;1}^{n}\delta (m_{i},\;p_{i})$$

The match score *S’* was calculated to assess the degree of agreement between an experimentally measured retention order {*m*_*i*_} of reference compounds and the predicted retention order {*p*_*i*_} of candidate compounds in the database. The match score was calculated by the same method as the calculation of prediction accuracy {*S*}.

### 2.6 Prediction by random forest

In addition to the RankSVM model, we constructed a Random Forest model for comparison. Model construction followed the same preprocessing steps as RankSVM. The training data consisted of retention time and molecular fingerprints extracted from the same datasets as RankSVM: the PredRet dataset and the Nematostella dataset, along with the reference compounds. MIX counting PCF reduced to 200 dimensions was used as explanatory variables. The script of Random Forest is available at the author’s repository: https://github.com/ShoheiNakamukai/RO_prediction_of_PNPA/tree/main.

### 2.7 Sample collection and measurement

A specimen of the marine sponge *Jaspis splendens* (de Laubenfels 1954) was collected by scuba diving at a depth of 15 m by an author (YI) at Tatsukushi Coast, Tosa-shimizu, Kochi, Japan (32°47′00.1″N, 132°51′06.1″E). The resulting extract was dissolved in methanol and subjected to LC/MS analysis with conditions in Supporting Table 1, available as supplementary data at *Bioinformatics Advances* online, for metabolite profiling.

## 3 Results

### 3.1 Evaluation and comparison of prediction models using different fingerprints for retention order training

We first conducted the training of retention order using Nematostella, PredRet, and the reference dataset. When we compared two prediction models using the MACCS binary fingerprint and the MACCS counting fingerprint as explanatory variables, the prediction accuracies were better for the counting fingerprint in all three datasets (Table 2, dataset 2a). This suggested that the occurrence counts of substructures could better grasp PNPA structures, although the improvement was little and not always significant. To maximize prediction potentials from structures, we integrated four types of fingerprints altogether (Xie *et al.* 2020, Breslin and Pham 2023). The prediction accuracy with the combined MIX fingerprint, however, did not improve results in all six conditions in its raw form (Table 2; dataset 2a).

**Table 2.** Prediction accuracy comparing the different explanatory variables for each training dataset (2a) and different number of variables for each training dataset (2b).^a^

2a				2b			
MACCS	Nematostella	PredRet	Nematostella + PredRet	Number of variables	Nematostella	PredRet	Nematostella + PredRet
Binary	0.59	0.72	0.68	50	0.68	0.75	0.77
Counting	0.75	0.73	0.74	100	0.68	0.81	0.78
MIX	Nematostella	PredRet	Nematostella + PredRet	200	0.69	0.79	0.904
Binary	0.65	0.75	0.72	300	0.67	0.8	0.899
Counting	0.79	0.72	0.66	400	0.67	0.78	0.87
PCF_MACCS	Nematostella	PredRet	Nematostella + PredRet	500	0.69		0.86
Binary	0.77	0.69	0.79	All	0.7	0.78	0.86
Counting	0.75	0.84	0.81	**2c**			
PCF_MIX	Nematostella	PredRet	Nematostella + PredRet	Training data	RankSVM	Randam Forest	
Binary	0.82	0.71	0.38	Nematostella	0.69	0.63	
Counting	0.7	0.78	0.86	PredRet	0.79	0.66	
				Nematostella + PredRet	0.904	0.63	

a 2a: The upper two tables show the result of direct use of all fingerprints, and the bottom two tables show the result of principal component fingerprints (PCF). 2b: The total number of variables in PredRet was 426, and the corresponding value for 500 is missing. 2c: Comparison of prediction accuracy between RankSVM and Random Forest models trained on PredRet, Nematostella, and combined datasets.

### 3.2 Addressing multicollinearity in fingerprints to improve prediction accuracy

We calculated the Variance Inflation Factor (VIF), an indicator of multicollinearity, for each training dataset (Yoo *et al.* 2014). The VIF values was high (exceeding 10) in all fingerprints, indicating a substantial level of multicollinearity (Supporting Table 2, available as supplementary data at *Bioinformatics Advances* online). To avoid the problem of multicollinearity, we applied principal component analysis (PCA) for each fingerprint (Zhang and Castelló 2017), and named the resulting explanatory variables principal component fingerprints (PCF). As shown in Table 2 (dataset 2a, bottom), using PCF improved prediction accuracy in most conditions. This highlights the effectiveness of PCF in enhancing the predictive capabilities of our model.

### 3.3 Enhancing prediction accuracy through variable selection on principal component fingerprints

To further improve the prediction accuracy, we conducted variable selection on PCF. In machine learning, variable selection can be broadly categorized into three methods: filter methods, wrapper methods, and embedded methods (Piles *et al.* 2021). In this study, RankSVM requires an extensive training period ranging from 12 to 24 hours, and we opted for the filter method due to its lower computational cost and overfitting risk. PC variables were stepwise chosen from 50 and 100 to 500 for the training as in Table 2 (dataset 2b). When using 200 PCF of Nematostella + PredRet, the prediction accuracy reached the highest value of 0.904, a slight improvement by variable reduction. Prediction accuracies were also improved for either of Nematostella or PredRet training data. We also explored the performance of an alternative machine learning method to verify the performance of RankSVM. We applied the Random Forest prediction for the best performing dataset in the RankSVM approach (Python package scikit-learn, version 1.1.2; see Section 2). This yielded a prediction accuracy of 0.63, markedly lower than the accuracy achieved by the RankSVM (0.904) (Table 2, dataset 2c), suggesting that the overall performance is comparable or superior to standard machine learning approaches.

### 3.4 Analysis of predicted retention orders for PNPAs against reference compounds

With this preparation, we predicted the retention order of each PNPA against the reference compounds (Table 3; Supporting Table 3, available as supplementary data at *Bioinformatics Advances* online). The retention order of vasopressin, which is composed only of proteinogenic amino acids, was predicted accurately, probably due to the training from the peptidomics dataset. Likewise, the high prediction accuracy was achieved for α-amanitin, which is essentially made up of proteinogenic amino acids modified with some oxygens. In contrast, the high prediction accuracy of epothilone B, which is partially biosynthesized by NRPS (Molnár *et al.* 2000), can be attributed to the training from the small molecule dataset.

**Table 3.** Prediction accuracy of each PNPA.

PNPA	Prediction accuracy
Vancomycin	0.91
[Arg8]-Vasopressin	1.00
α-Amanitin	1.00
Bacitracin	0.64
Daptomycin	0.77
Epothilone B	1.00
Gramicidin A	0.91
Actinomycin D	0.95
Cyclosporine A	0.95
AVERAGE	0.904

### 3.5 Dataset splitting and proportions on prediction accuracy

To assess the effect of dataset splitting, we divided the Nematostella dataset from 5 to 10 subsets and the PredRet dataset from 4 to 8, and trained for each configuration of Nematostella_10 and PredRet_8. When each dataset was independently used, prediction accuracy did not improve. When they were combined, however, improvement was substantial, and revealed that the splitting of the Nematostella dataset did not contribute to the improvement (Table 4).

**Table 4.** Impact of dataset splitting on prediction accuracy.^a^

	Before splitting	After splitting
Nematostella_10	0.7	0.69
PredRet_8	0.78	0.77
PredRet_8 + Nematostella	0.86	0.914
PredRet + Nematostella_10	0.86	0.86
PredRet_8 + Nematostella_10	0.86	0.88

a The Nematostella dataset was divided into 10 subsets (Nematostella_10) and the PredRet dataset was 8 subsets (PredRet_8).

The improvement by splitting PredRet dataset presumably came from its heavy overlap in RT values due to its shorter chromatographic runs. The PredRet dataset includes a wide range of small molecules with varying RTs, but the majority was measured within a short retention time. High RT overlaps lead to learning ambiguity because the ranking kernel considers the ordering only (there is no notion of ties). By splitting the PredRet dataset, the RT pairs with significant overlaps were distributed across multiple subsets, effectively reducing the ambiguous pairwise comparisons. Thereby, the model’s capacity to learn accurate retention orders was enhanced. In addition, splitting the dataset increased the number of cross-validation folds which improved the robustness of training and testing processes, as the model was tested on a broader variety of data distributions (Kohavi 1995).

### 3.6 Identifying key variables influencing retention order prediction using SHAP values

**Figure 2.**
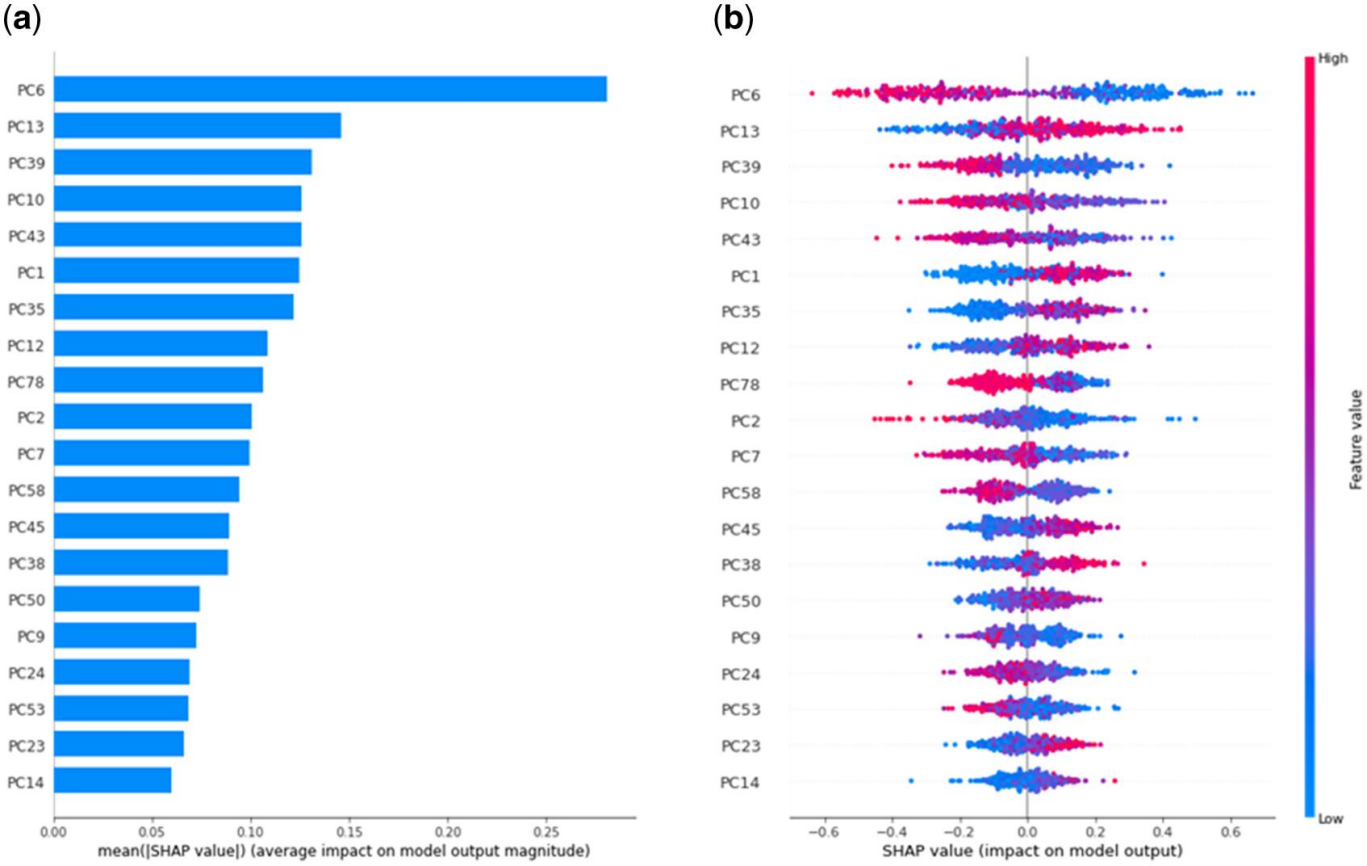
SHAP values for the prediction model using PCF. (a) The top 20 variables with high contribution to the prediction model. (b) The dependence plot of the top 20 variables with high contribution to the prediction model. The positive SHAP values indicate positive correlations with each PC and the negative SHAP values indicate negative correlations with each PC (positive correlation refers to when the retention order is the same as the order of input of two compounds).

To explore the key variables influencing retention order prediction, we calculated SHAP (SHapley Additive exPlanations) values for the prediction model (Lundberg and Lee 2017, Wojtuch *et al.* 2021). The SHAP calculation assigns a contribution value (SHAP value) to each variable in a particular prediction to reveal its importance. We examined highly contributing variables for the case of MIX counting PCF of PredRet + Nematostella dataset (Fig. 2a). In this case, PC6 showed the largest contribution, whose structural interpretation is methyl groups (explained in the next subsection).

**Figure 3.**
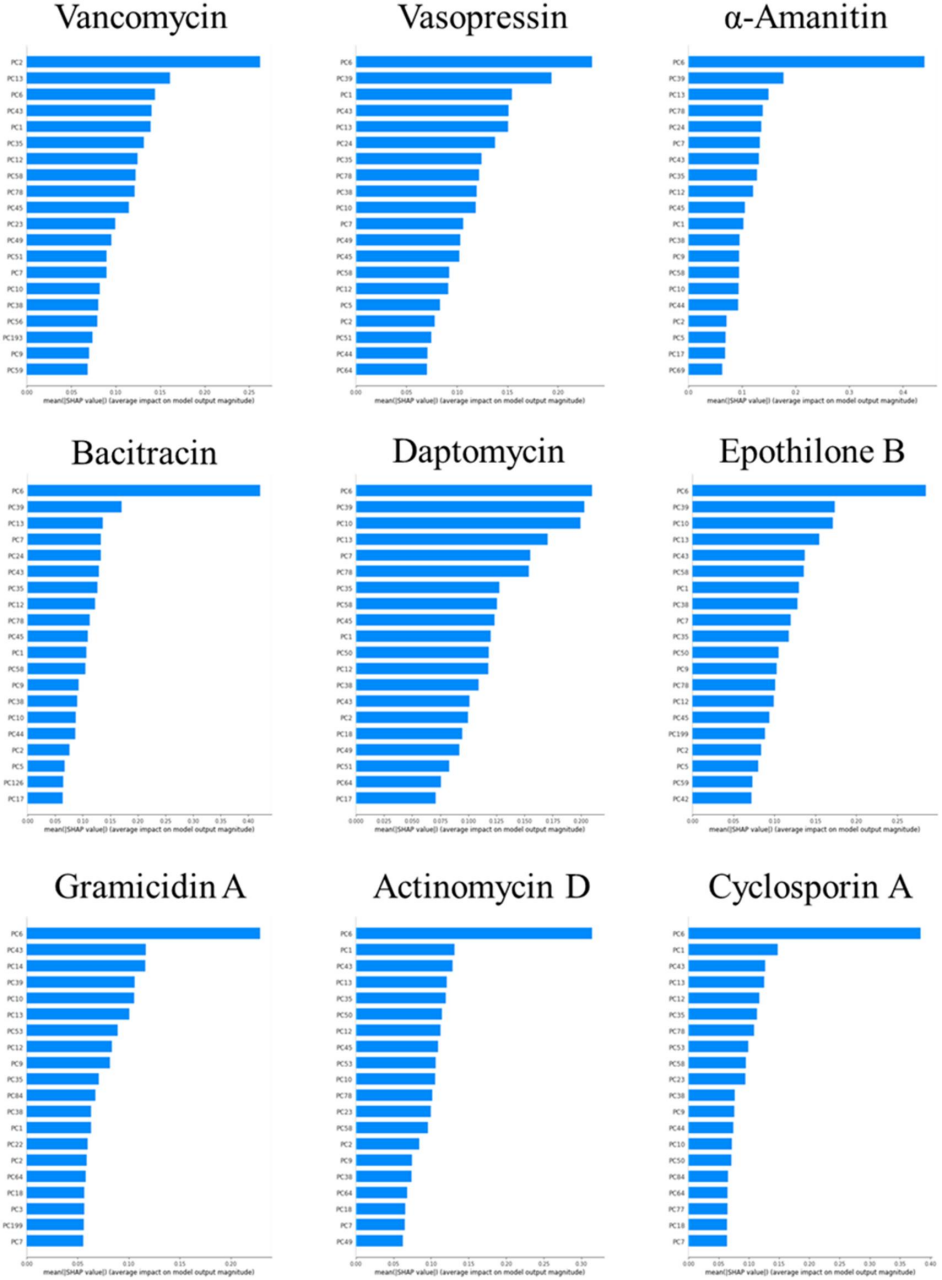
The top 20 SHAP values for each PNPA.

To analyze the crucial variables for each PNPA, we examined the SHAP values for each PNPA (Fig. 3). PC6 had the highest SHAP values in all PNPAs except for vancomycin. Among the five PNPAs ([Arg8]-vasopressin, α-amanitin, bacitracin, daptomycin, and epothilone B) with intermediate retention times, PC39 had the second-highest SHAP value. Furthermore, among the three PNPAs (gramicidin A, actinomycin D, and cyclosporine A) with later retention times, PC1 exhibited higher SHAP value.

### 3.7 Correlation of substructures with principal components and their impact on retention time prediction

To investigate which substructures are correlated with each PC, principal component loadings (Supporting Table 4, available as supplementary data at *Bioinformatics Advances* online), an indicator to quantify each variable’s contribution to the principal components, were investigated. The principal component loadings for each PC were calculated and the top five absolute values of SMART pattern of MACCS keys (due to the ease of interpretation of the MACCS fingerprints, we omitted the other three fingerprints) were shown in the Table 5. According to the results, PC6 of the highest SHAP value was associated with descriptors related to methyl groups. On the other hand, the second highest PC13 and the third highest PC39 were associated with ring structures and halogens/hetero atoms, respectively.

**Table 5.** Top five principal component loadings for each PC.^a^

PC1		PC2	
SMART pattern	CR	SMART pattern	CR
NA(A)A	0.0816	OC(C)C	0.1154
QN	0.0808	6M ring	0.1139
C=O	0.0797	6M ring	0.1139
C-N	0.0796	Ring	0.1106
O=A	0.0794	A$A! O	0.1062

PC6		PC13	
SMART pattern	CR	SMART pattern	CR
CH3ACH2A	0.1205	CH3CH2A	−0.1211
CH3CH2A	0.1149	Anot%A%Anot%A	−0.0969
AQ(A)A	0.0857	AQ(A)A	−0.0753
CH2QCH2	0.0767	A! A$A! A	0.0748
CH3	0.0665	QAAAA@1	−0.0724

PC39	
SMART pattern	CR
X	0.1092
CH2QCH2	−0.0835
A$A! N	0.0834
AQ(A)A	−0.0745
X! A$A	0.0743

a CR, contribution rate; A, any atom; Q, hetero atom; X, halogen; -, single bond; =, double bond; $, ring bond; !, chain or non-ring bond; %, aromatic bond; @1, ring linkage to the first atom.

**Figure 4.**
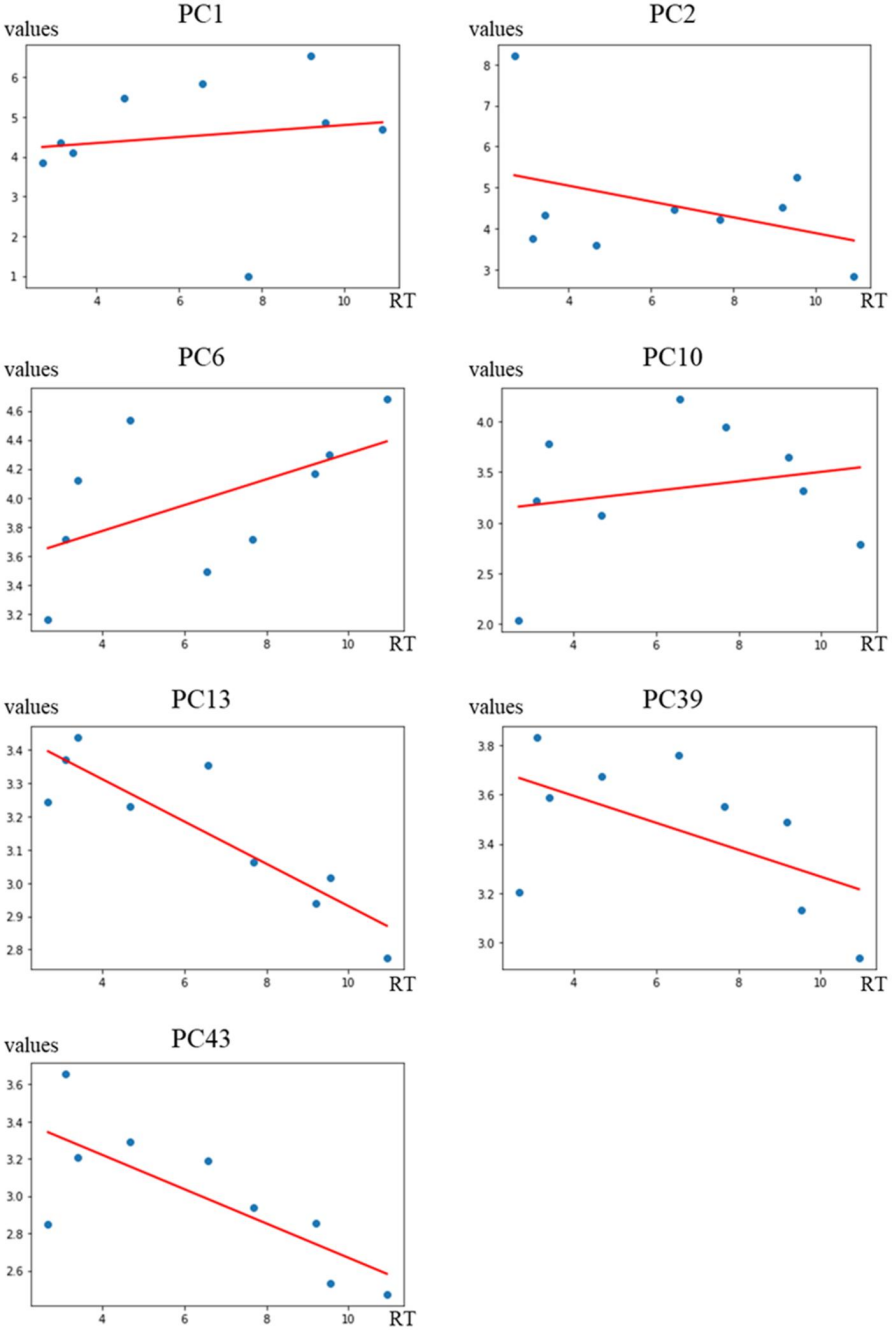
Relationships between retention time of PNPA and each PCF.

Figure 4 shows the relationship between PCF and the retention time of PNPA. PC6 showed positive, and PC13 and PC39 showed negative correlations with retention time. These findings are corresponding with that hydrophobic substructures are positively correlated with retention time, and hydrophilic substructures are negatively correlated with retention time. It is note that PC6 of bacitracin and daptomycin greatly deviated from the regression line (Fig. 4). The low prediction accuracy of these two PNPAs was thought to be caused by these deviations.

### 3.8 Visual assessment of the representativeness of standard PNPA using PCA

**Figure 5.**
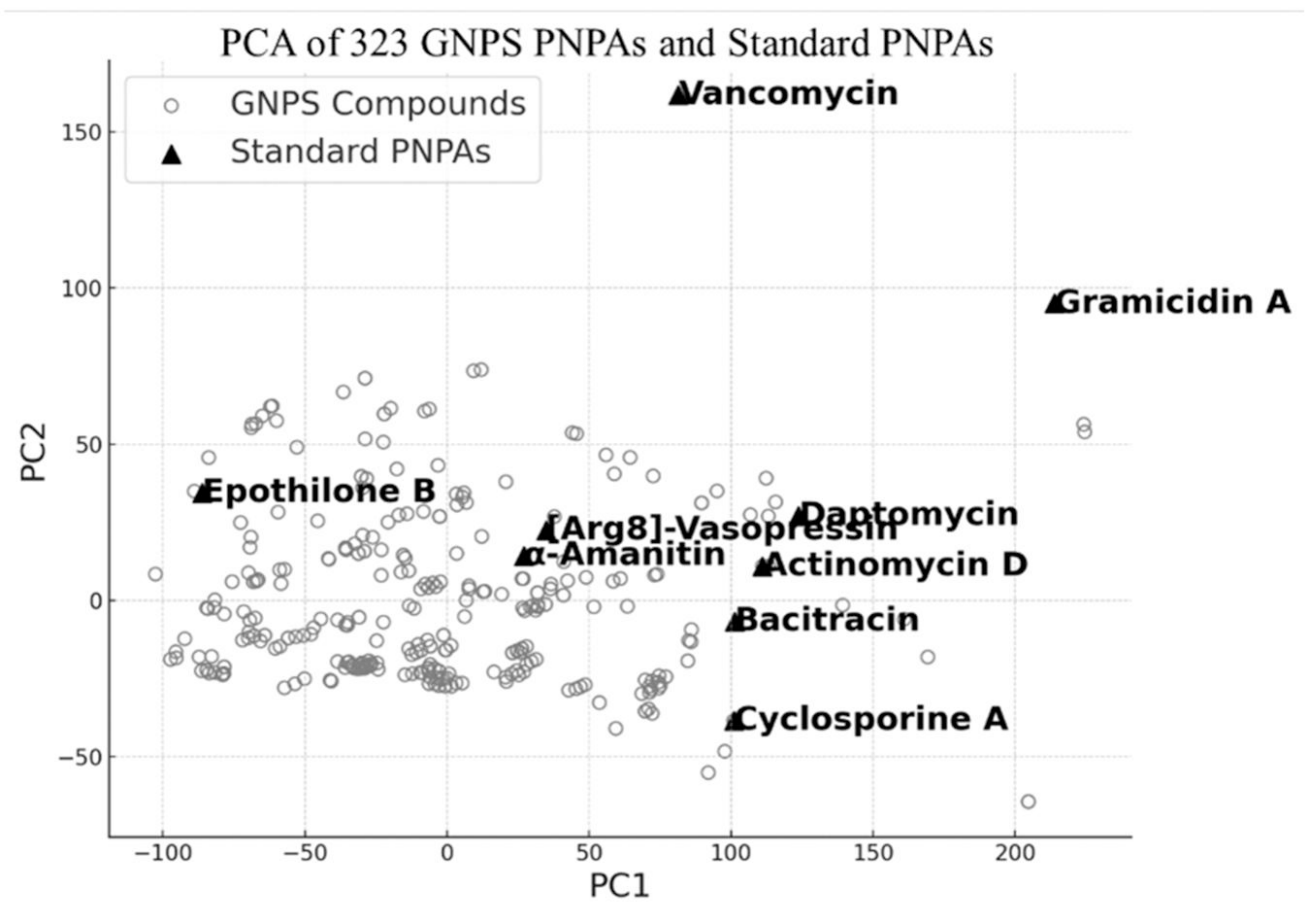
Principal component analysis (PCA) of 323 PNPA structures from GNPS and nine standard PNPAs. PCA was performed on MIX fingerprints calculated from the 323 PNPA compounds retrieved from GNPS and the nine selected standard PNPAs. Gray circles represent the GNPS-derived PNPAs, and black triangles represent the standard PNPAs used in this study.

To assess how well the nine selected standard PNPAs represent the structural diversity of PNPAs, we compared them with available 323 PNPAs from Global Natural Products Social Molecular Networking (GNPS; http://gnps.ucsd.edu) (Wang *et al.* 2016) (Supporting Table 7). Principal component analysis (PCA) was performed on MIX fingerprints calculated from the 323 GNPS- and the nine standard PNPAs. The resulting PCA plot (Fig. 5) shows that the standard PNPAs are broadly distributed across the chemical space occupied by the GNPS PNPAs. This suggests that the selected standard PNPAs represent structural diversity of the overall chemical space of PNPAs for model training.

### 3.9 Application of retention order prediction model for compound identification in natural product databases

To assess the practicality of the retention order prediction for compound identification, we applied this model to two natural product databases, COCONUT (Sorokina *et al.* 2021) and NPEdia (Tomiki *et al.* 2006). Specifically, we used the latest 2021 version of the latter database, which is available on Zenodo (https://zenodo.org/search?q=parent.id%3A3547717&f=allversions%3Atrue&l=list&p=1&s=10&sort=version). First, *m*/*z* of the test PNPA was searched on each database with a tolerance of ±0.5. Then the retention order for each hitting compound was predicted using our model (MIX counting PCF of Nematostella + PredRet), and the match score *S’* was calculated (see Section 2.5). If the RT order between the measured compound and a hitting compound was very different, it means that we can narrow down the target compound efficiently only from structural information.

**Figure 6.**
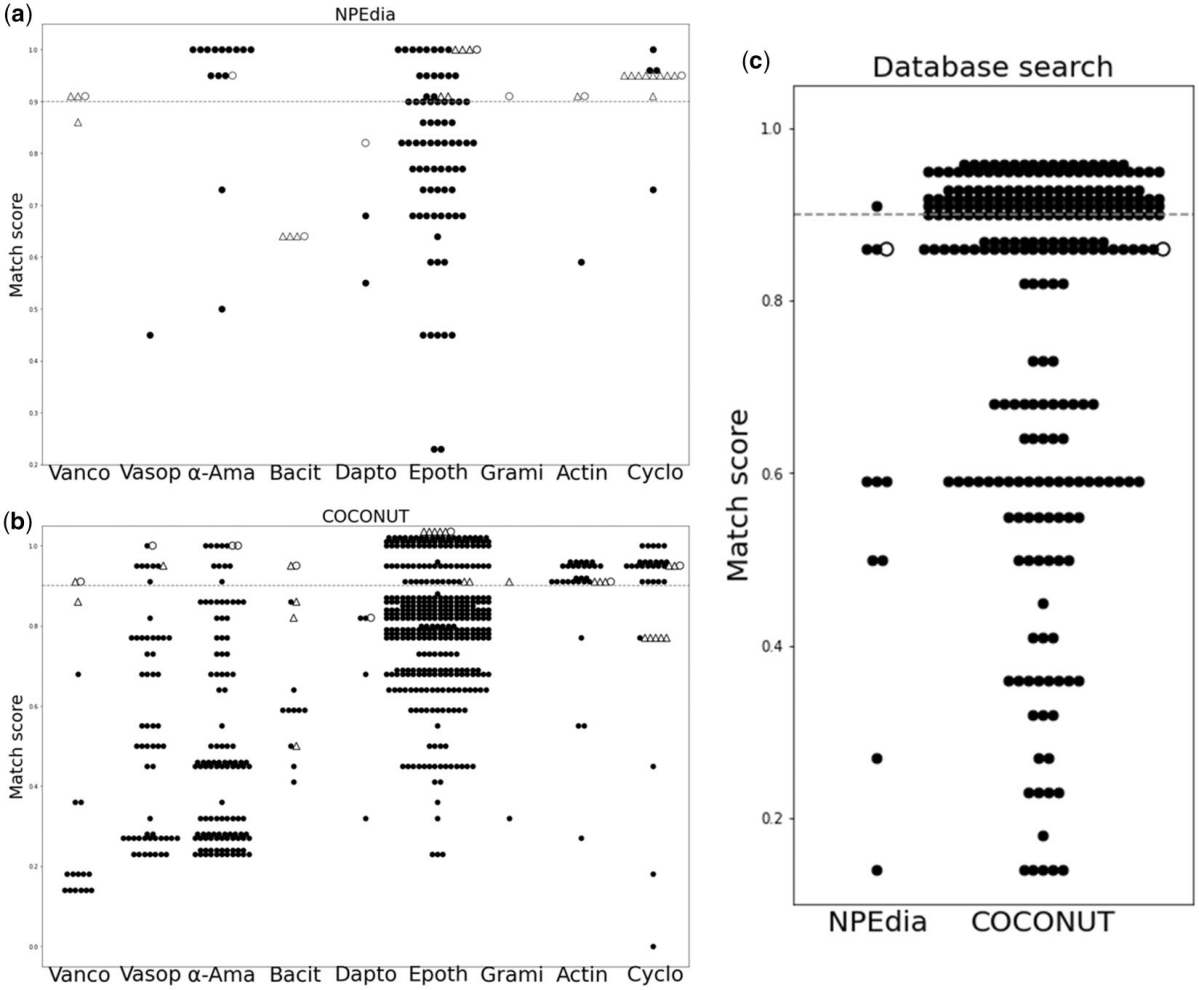
Identification of test compounds using the retention order prediction model. (a) NPEdia database search results. (b) COCONUT database search results. (c) Application of retention order prediction to compounds derived from *Jaspis splendens*. In (a) and (b), the dashed line indicates the match score threshold of 0.9. White circles and triangles represent the test compounds and their analogs, respectively. In (c), the compound was searched against COCONUT and NPEdia using *m*/*z* and retention time information. Retention order prediction enabled narrowing down the candidate structures, and the true structure, Jaspsamide, is indicated by the white circles. Vanco, vancomycin; Vasop, vasopressin; α-Ama, α-amanitin; Bacit, bacitracin; Dapto, daptomycin; Epoth, epothilone B; Grami, gramicidin A; Actin, actinomycin D; Cyclo, cyclosporine A.

We set the RT threshold for isomers as 0.9 (dashed line in Fig. 6) or higher. In the COCONUT search results, all the test PNPAs except for gramicidin A were contained. For vancomycin, bacitracin, and gramicidin A, only their analogs showed matching scores higher than 0.9. For vasopressin and α-amanitin, many candidates were excluded efficiently by the filtering. However, for daptomycin, epothilone B, actinomycin D and cyclosporin A, filtering did not work efficiently (Fig. 6a, Table 6; Supporting Table 5, available as supplementary data at *Bioinformatics Advances* online). In the NPEdia search results, all the test PNPAs except for vasopressin were contained. For vancomycin, bacitracin, and gramicidin A, only their analogs were selected. In addition, daptomycin could be uniquely identified as the highest hit. Actinomycin D was also selected with a score higher than 0.9 (Fig. 6b, Table 6; Supporting Table 6, available as supplementary data at *Bioinformatics Advances* online).

**Table 6.** Results of database search for test PNPA.^a^

COCONUT				
Test PNPA	Total hits (*m*/*z *± 0.5)	Presence of test PNPA	Match score ≥ 0.9	Analog count (including test PNPA)
Vancomycin	17	○	2	3 (CNP0183742: Vancomycin, CNP0326659: Dihydrobalhimycin, CNP0338260)
Vasopressin	57	○	9	2 (CNP0165945: [Arg]-Vasopressin, CNP0398499)
α-Amanitin	115	○	12	2 (CNP0135846: α-Amanitin, CNP0426433: α-Amanitin)
Bacitracin	15	○	2	5 (CNP0339579, CNP0250680, etc.)
Daptomycin	5	○	0	1 (CNP0282041: Daptomycin)
Epothilone B	381	○	99	8 (CNP0147990: Epothilone B, CNP0078189, etc.)
Gramicidin A	2	×	1	1 (CNP0336908)
Actinomycin D	32	○	28	4 (CNP0226661: Actinomycin D, CNP0081136: C-Demethylactinomycin, CNP0086084: Actinomycin Y9, CNP034756)
Cyclosporin A	36	○	27	8 (CNP0187739: Cyclosporin A, CNP0458389, etc.)

NPEdia				
Test PNPA	Total hits (*m*/*z *± 0.5)	Presence of test PNPA	Match score ≥ 0.9	Analog count (including test PNPA)
Vancomycin	4	○	3	4 (FSL0943: Vancomycin, FSL0944, A51568B, Antibiotic PA 45052B)
Vasopressin	1	×	0	0
α-Amanitin	15	○	13	1 (α-Amanitin)
Bacitracin	4	○	0	4 (FSL0383: Bacitracin, Ayfivin, etc.)
Daptomycin	3	○	0	1 (A 21978 C0: Daptomycin)
Epothilone B	79	○	22	6 (Epothilon B, 9-Hydroxyepothilone D, 11-Hydroxyepothilone D, etc.)
Gramicidin A	1	○	1	1 (Gramicidin A)
Actinomycin D	3	○	2	2 (FSL0334: Actinomycin D, Aurantin II)
Cyclosporin A	14	○	13	10 (Cyclosporin A, Cyclosporin-I, etc.)

a In analog count, hit compounds are written as COCONUT ID and NPEdia name, respectively.

### 3.10 Proof of concept study of retention order prediction

We applied the retention order prediction model to unknown compounds from a specimen of marine sponge Jaspis splendens collected in a local seabed in Japan (see Section 2). LC/MS data were obtained from the sponge extract prepared for metabolite profiling, providing precursor *m*/*z* and retention time information. Candidate structures retrieved by mass filtering based on molecular weights (±0.5 Da) from the natural product database NPEdia were evaluated by retention order prediction model (Supporting Table 8). Figure 6c illustrates the match scores of all retrieved compounds. In NPEdia, 11 candidate structures were retrieved, and the retention order prediction selected four candidates with higher match scores. Among these was Jaspamide, a peptide previously reported from *Jaspis splendens* (Zabriskie *et al.* 1986). To verify that the correct structure was prioritized by the retention order prediction, previously reported MS/MS spectrum of Jaspamide was searched in literature and good match was confirmed with the measured MS/MS spectrum (Ramanjooloo *et al.* 2023). This case demonstrates that the retention order prediction can effectively support structure annotation in natural product discovery.

## 4 Conclusion

In this study, we assessed the prediction accuracy of the machine learning model by comparing the predicted retention order for PNPA with the measured retention order from small-molecule and peptide datasets. We achieved a prediction accuracy exceeding 0.9 for PNPAs, comparable to the previous achievement for small molecules (Bach *et al.* 2018). This is noteworthy because the properties of small molecules are very different from those of PNPA, and the high accuracy was achieved through PCA and dataset splitting. The key substructures for the prediction were methyl groups, ring structures, and halogens. This indicates that we need to measure more metabolites and peptides with these structures for even better predictions.

## Supplementary Material

vbaf246_Supplementary_Data

## Data Availability

The data underlying this article are available in the DNA Data Bank of Japan (DDBJ) at https://www.ddbj.nig.ac.jp/bioproject/, and can be accessed with BioProject ID PRJDB35568 and six BioSample entries SAMD01592756-SAMD01592766.
