# Supplementary material for: Retention order prediction of peptides containing non-proteinogenic amino acids
Source: Bioinform Adv. 2025 Oct 8;5(1):vbaf246. doi: 10.1093/bioadv/vbaf246 (PMC12643230; doi:10.1093/bioadv/vbaf246)
Supplement: vbaf246_Supplementary_Data [file vbaf246_supplementary_data.zip › Supplement_Data.zip!/Supporting Section.docx]

**Supporting**

**Section 1. Selection and preparation of datasets for retention order prediction experiments**

We selected four systems (Eawag_XBridgeC18 (317 compounds), FEM_long (281 compounds), RIKEN (181 compounds), UFZ_Phenomenex (192 compounds)) for the main experiments and they were named 'PredRet'. In addition, other five systems (LIFE_old (183 compounds), LIFE_new (173 compounds), FEM_lipids (86 compounds), FEM_orbitrap_plasma (120 compounds), SMRT (247 compounds)) were selected for the control experiment and they were named 'PredRet_2'. These datasets contain flavonoids, alkaloids, terpenoids, amino acids, glucosinolates, and others. The mass RAW data of peptides and single-cell RNA sequencing data were downloaded from the ProteomeXchange Consortium via jPOST with the data set identifier PXD030145 (Hayakawa et al., 2022). From this dataset, three datasets Nematostella_n1.raw, Nematostella_n2.raw, and Nematostella_n3.raw were selected as training data for the main experiment and they were named “Nematostella”. In addition, other three datasets Bolinopsis_n1.raw, Bolinopsis_n2.raw, Bolinopsis_n3.raw were selected for the control experiment and they were named “Bolinopsis”. The extraction of mass information from the Raw data was performed using MaxQuant (v.2.2.0). The search parameters were as follows: 4.5 ppm for precursor tolerance, 0.5 Da for fragment tolerance, no fixed modifications, oxidation of methionine, pyroglutamation from glutamic acid of glutamine and amidation at peptide or protein C termini. From the extracted mass information, the sequence data and retention time data of 787 peptides were obtained from Nematostella. These peptides were sorted in order of retention time, and they were sequentially assigned into five groups (Nematostella_1, Nematostella_2, Nematostella_3, Nematostella_4 and Nematostella_5) in such a way that the retention time distribution was made uniform among each group. Additionally, 693 proteinogenic peptides were obtained from Bolinopsis and they were assigned into five groups (Bolinopsis_1, Bolinopsis_2, Bolinopsis_3, Bolinopsis_4, Bolinopsis_5) in the same way.

**Section 2. Molecular fingerprinting and principal component analysis**

We represented the molecular structure by binary and counting fingerprints. We calculated four types of fingerprints (MACCS, extended standards, graph and circular) by using rcdk R-package (Guha, 2007) from SMILES, which were transformed from the InChI in PredRet compounds and from the amino acid sequences in peptides by using RDkit. The SMARTS patterns utilized for the calculation of MACCS fingerprint were shown in the previous paper (Bach et al., 2018). The four types of fingerprints were all combined and bit strings with a usage frequency of 10% or less were deleted for the purpose of simplifying the model, speeding up the calculation, and improving the prediction accuracy (Ji et al., 2020), and we named the obtained fingerprints MIX fingerprint.

Principal component analysis (PCA) was performed for the preprocessing of fingerprints. First, we combined the training datasets used for each experiment and normalization was performed on it. For the test dataset, each variable was divided by the maximum value of the corresponding variable in the training dataset and the normalized values in the test dataset that exceeded 1 were set to 1. PCA was performed on each normalized training dataset and its transformation matrices were used to transform the test datasets.

**Section 3. Impact of combined training datasets on retention order prediction accuracy**

To ensure an equal number of training data, we applied the same training process to the combined dataset of Nematostella and Bolinopsis (Nematostella + Bolinopsis, as described in the methods section) and to the combined datasets of PredRet and PredRet_2 (PredRet + PredRet_2), and we performed retention order predictions. As shown in Table 7, the prediction accuracies for both datasets, Nematostella + Bolinopsis and PredRet + PredRet_2, were lower than that of Nematostella + PredRet. Consequently, it was demonstrated that utilizing both peptidomics and small molecules as training data leads to higher prediction accuracy.

| **Table S1**: Prediction accuracy for each training dataset | |
| --- | --- |
| Training data | prediction accuracy |
| Bolinopsis | 0.59 |
| PredRet_2 | 0.82 |
| Bolinopsis + PredRet_2 | 0.81 |
| Nematostella + Bolinopsis | 0.78 |
| PredRet + PredRet_2 | 0.60 |

Bach, E., Szedmak, S., Brouard, C., Böcker, S., & Rousu, J. (2018). Liquid-chromatography retention order prediction for metabolite identification. *Bioinformatics*, *34*(17), i875–i883. https://doi.org/10.1093/bioinformatics/bty590

Guha, R. (2007). Chemical Informatics Functionality in R. *Journal of Statistical Software* , *18*(5). http://www.jstatsoft.org/

Hayakawa, E., Guzman, C., Horiguchi, O., Kawano, C., Shiraishi, A., Mohri, K., Lin, M. F., Nakamura, R., Nakamura, R., Kawai, E., Komoto, S., Jokura, K., Shiba, K., Shigenobu, S., Satake, H., Inaba, K., & Watanabe, H. (2022). Mass spectrometry of short peptides reveals common features of metazoan peptidergic neurons. *Nature Ecology and Evolution*, *6*(10), 1438–1448. https://doi.org/10.1038/s41559-022-01835-7

Ji, H., Deng, H., Lu, H., & Zhang, Z. (2020). Predicting a Molecular Fingerprint from an Electron Ionization Mass Spectrum with Deep Neural Networks. *Analytical Chemistry*, *92*(13), 8649–8653. https://doi.org/10.1021/acs.analchem.0c01450
